# Assessment of Quality of Life in Patients With Upper Aerodigestive Tract Cancer

**DOI:** 10.7759/cureus.100219

**Published:** 2025-12-27

**Authors:** Diana Almendariz Ramos, Marco Antonio Mendez Saenz, Jose Luis Trevino Gonzalez

**Affiliations:** 1 Otolaryngology - Head and Neck Surgery, Hospital Universitario Dr. José Eleuterio González, Monterrey, MEX

**Keywords:** head and neck cancer, health related quality of life, quality of life, social emotional well-being, upper aerodigestive tract cancer

## Abstract

Introduction

Head and neck cancer can compromise swallowing, breathing, and speech, and may markedly reduce health-related quality of life (HRQOL), particularly because it often involves visible structures. This study evaluated HRQOL in Mexican patients with upper aerodigestive tract (UADT) cancer before and after unimodal or multimodal treatment using the Functional Assessment of Cancer Therapy-Head & Neck (FACT-H&N) questionnaire.

Materials and methods

This was an observational, analytical, longitudinal study of 68 patients. The validated Spanish version of the FACT-H&N was administered to patients treated at the “Dr. José Eleuterio González” University Hospital from July 2024 through July 2025. Demographic characteristics, tumor site and stage, histology, and treatment were recorded.

Results

A total of 68 patients with head and neck cancer were included (mean age 60.2 ± 12.7 years); 34 patients (50.0%) had at least one comorbidity. Moderately differentiated keratinizing squamous cell carcinoma predominated, occurring in 30 patients (44.1%), and most tumors presented at advanced stages (III-IV). The most frequent primary sites were the oral cavity in 29 patients (42.6%) and the larynx in 21 patients (30.9%). Treatment was predominantly multimodal: surgery was used alone or combined with chemotherapy and/or radiotherapy, and concurrent chemoradiotherapy was used in 11 patients (16.7%). Postoperatively, 33 patients (48.6%) had no complications, 19 (27.9%) developed complications, and in 16 (23.5%), this information was not recorded. Regarding HRQOL, Physical Well-Being was the lowest domain at baseline and at three months, whereas Social/Family Well-Being was the highest. Emotional Well-Being showed the greatest decline and remained low, while the Head & Neck (H&N) subscale decreased at one month and returned to baseline by three months; the global score did not change significantly. By treatment modality, surgery was associated with better Functional Well-Being and higher Social/Family Well-Being (both at baseline and three months) but with lower Emotional Well-Being at three months; non-surgical patients had better Physical Well-Being at three months. Radiotherapy did not alter the overall trajectory, although H&N subscale scores worsened after radiotherapy and at three months. Chemotherapy did not change the overall course but was associated with higher Physical Well-Being and lower Emotional Well-Being and H&N subscale scores.

Conclusion

HRQOL in patients with UADT cancer may improve in specific domains after treatment; however, overall scores may remain unchanged, highlighting the need for adjunct supportive interventions and follow-up protocols that extend beyond oncologic disease control.

## Introduction

Head and neck cancers (HNCs) comprise a heterogeneous group of neoplasms arising between the skull base and the thoracic inlet--a region of considerable anatomic complexity that includes the salivary and thyroid/parathyroid glands, the middle ear, and a dense neurovascular network. Given this intricate compartmental anatomy and the proximity to critical aerodigestive and neurovascular structures, cross-sectional imaging (CT and MRI) with standardized, site-specific reporting is essential for accurate staging, treatment planning, and post-treatment assessment [1]. Clinically and epidemiologically, these tumors are commonly organized around the upper aerodigestive tract (UADT), which includes digestive components (oral cavity, oropharynx, hypopharynx, upper esophageal sphincter, and cervical esophagus) and respiratory components (nasal cavity and paranasal sinuses, nasopharynx, larynx, and trachea). Most HNCs originate from the mucosal epithelium of the UADT and are squamous cell carcinomas, whereas the remainder arise from other cervicofacial tissues such as thyroid, salivary glands, skin, and bone/cartilage. Definitions vary across authorities, but the UADT framework remains widely used in research, imaging interpretation, and clinical practice [1-3].

Globally, major mucosal HNC subsites impose a substantial cancer burden. Cancers of the lip and oral cavity rank sixteenth and laryngeal cancer ranks twentieth in worldwide incidence, with marked geographic heterogeneity and consistently higher incidence in men than in women [2,4,5]. Tobacco and alcohol act synergistically as major etiologic drivers across many subsites. Oncogenic viruses also shape disease distribution--human papillomavirus (HPV), particularly in oropharyngeal cancer and Epstein-Barr virus in nasopharyngeal cancer--while betel-quid chewing further increases risk in parts of South and Southeast Asia [2,6-9]. Within the UADT framework, cancers of the lip, oral cavity, and pharynx (LOCP) comprise a major proportion of mucosal head and neck malignancies and account for approximately one in 25 of all new cancer diagnoses and cancer deaths worldwide, reflecting a substantial public health burden [6-9]. In many high-income settings, HPV-positive oropharyngeal squamous cell carcinoma has increased over time even as tobacco-related sites have shown declines, underscoring evolving epidemiology and prevention priorities [4].

Despite advances in combined-modality therapy--surgery, radiotherapy (including intensity-modulated techniques), and systemic therapy--many patients still present with advanced clinical stage, and nodal disease remains a major determinant of outcome. Careful imaging-based assessment of primary tumor extent, nodal metastases, and treatment response is therefore crucial [1,2]. Beyond oncologic outcomes, symptom burden and emotional distress can substantially degrade health-related quality of life (HRQOL) in HNC. Pain, mucositis, xerostomia, taste and smell disturbances, dysphagia, and nutrition-impact symptom clusters are common during and after treatment, justifying systematic HRQOL assessment in both clinical care and research. Moreover, mixed-method evidence shows that HRQOL is compromised across physical, functional, emotional, and social domains from diagnosis through early survivorship; patients report swallowing and speech difficulties, altered taste, xerostomia, pain, fatigue, body-image concerns, financial strain, caregiver burden, and unmet informational needs--issues that may be underrecognized by quantitative scales alone. These findings support collecting baseline and serial HRQOL measures to trigger timely nutritional, speech-language, and psychosocial interventions and to inform shared decision-making [10].

The primary objective of this study was to evaluate HRQOL in Mexican patients with UADT cancer before and after unimodal or multimodal treatment using the Functional Assessment of Cancer Therapy-Head & Neck (FACT-H&N, version 4.0), comparing each patient’s FACT-H&N scores at three time points: pre-treatment, end of treatment, and three months post-treatment. A secondary objective was to compare HRQOL trajectories across treatment groups.

## Materials and methods

Study design and setting

We conducted an observational, analytical, longitudinal study of 68 consecutive patients managed at the Otorhinolaryngology and Head and Neck Surgery Service of the “Dr. José Eleuterio González” University Hospital in Monterrey, Nuevo León, Mexico, from July 2024 through July 2025. All participants provided written informed consent.

Eligibility criteria

Inclusion criteria were age ≥18 years, either gender, care at the study unit, and histopathologically confirmed HNC. Exclusion criteria were malignancies outside the head and neck region, refusal to complete questionnaires, suspected but unconfirmed HNC, absence of oncologic treatment, and age <18 years.

Variables and time points 

We collected demographic data (age, sex, educational attainment, marital status), histopathologic diagnosis, primary tumor site, clinical stage, and treatment modality. HRQOL was measured at three prespecified time points: baseline (pre-treatment), within the first month after completion of treatment, and at three months post-treatment.

HRQOL instrument

HRQOL was assessed with the FACT-H&N version 4.0. The instrument comprises 39 items (27 core/general and 12 head-and-neck-specific) across four primary subscales--Physical, Social/Family, Emotional, and Functional Well-Being--plus the H&N subscale. Items are rated on a five-point Likert scale (0-4); domain and total scores are calculated per FACIT scoring guidelines, yielding a total score from 0 to 156, with higher scores indicating better HRQOL. The FACT-H&N was used with permission from FACIT.org and has been recommended for integration into clinical protocols for HNC patient-reported outcomes [10,11].

The questionnaire was administered during scheduled follow-up visits in a face-to-face setting. Eligible patients provided written informed consent after the study procedures and the FACT-H&N v4.0 were explained (or, if they were unable to sign, consent was obtained from a legally authorized representative). Patients then completed the FACT-H&N at the planned time points; those requiring assistance, including due to disability-related limitations, received standardized support from a member of the research team (e.g., reading items and recording responses) without interpreting questions or influencing answers.

Ethics, sample size, and oversight

The study upheld confidentiality standards and was approved by the Ethics Committee of the University Hospital of the Universidad Autónoma de Nuevo León, which issued approval OT24-00006. The target sample size (≥65 patients) was calculated to estimate a mean in a large population (expected mean 55.07, SD 12.38) with two-sided α=0.05 and 97.5% power, using parameters derived from the literature [10]. The final analytic cohort included 68 patients.

Statistical analysis

Descriptive statistics included frequencies and percentages for categorical variables and means (SD) or medians (IQR) for continuous variables, as appropriate. Normality was assessed with the Kolmogorov-Smirnov test. Inferential analyses employed χ² or Fisher’s exact test for independent categorical variables; McNemar’s test for paired categorical comparisons; Student’s t-test or Mann-Whitney U test for independent continuous variables; and paired t-test or Wilcoxon signed-rank test for within-subject continuous comparisons. All tests were two-sided with statistical significance set at p<0.05; 95% confidence intervals (CI) are reported where applicable. Analyses were performed using SPSS version 25 (IBM Corp., Armonk, NY, USA).

Primary and secondary outcomes

The primary outcome was the change in the FACT-H&N total and subscale scores across the three time points. Secondary analyses compared HRQOL trajectories between treatment groups (e.g., surgery alone, radiotherapy, chemotherapy, and multimodality combinations).

## Results

A total of 68 patients were included (mean age 60.15 ± 12.74 years), most of whom were between 50 and 70 years of age; 51 patients (75.0%) were men. Basic/primary education predominated, and 50 patients (73.5%) were married or in a consensual union. Most patients were employed, with 37 patients (54.4%) in active employment. Half of the cohort, 34 patients (50.0%), had at least one comorbidity--most commonly hypertension and type 2 diabetes mellitus (Table 1). 

**Table 1:** Sociodemographic data Data are expressed as frequencies and percentages in parentheses for categorical variables and mean with standard deviation for numerical variables.

Variable	N (%)
*Gender *(male)	51 (75)
Age	60.15 ± 12.74
Schooling	
No	3 (4.4)
Primary	25 (36.8)
Secondary school	19 (27.9)
High school	8 (11.8)
Degree	11 (16.2)
Graduate	2 (2.9)
Occupation	
Employee	37 (54.4)
Unemployed	17 (25)
Retired	14 (20.6)
Marital status	
Single	11 (16.2)
Free union	9 (13.2)
Married	41 (60.3)
Divorced	4 (5.9)
Widowed	3 (4.4)

Moderately differentiated keratinizing squamous cell carcinoma was the most frequent diagnosis, occurring in 30 patients (44.1%), followed by other squamous cell carcinoma variants and less common neoplasms. The oral cavity was the most commonly involved site, affecting 29 patients (42.6%), followed by the larynx in 21 patients (30.9%). Advanced clinical stage (III-IV) predominated, with few patients presenting with distant metastasis at diagnosis (Table 2). Management was predominantly surgical, either alone or in combination with systemic and/or radiation therapy. Specifically, surgery was combined with chemotherapy in 1 patient (1.5%), radiotherapy in 11 patients (16.2%), and both chemotherapy and radiotherapy in 28 patients (41.2%). In addition, 11 patients (16.7%) received concurrent chemoradiotherapy without surgery (Table 2).

**Table 2:** Oncological profile Data are expressed as frequencies and percentages in parentheses for categorical variables, while numerical variables are expressed with median and interquartile range in square brackets. ^a^ It includes SCC variants (non-keratinizing; well/moderately/poorly differentiated; with/without invasion; sinonasals) and aporadic variants like parotid Warthin-like mucoepidermoid carcinoma, parotid intraductal carcinoma, epithelial–myoepithelial carcinoma ex pleomorphic adenoma, neuroendocrine carcinoma, undifferentiated carcinoma of the nasal cavity, non-neuroendocrine sinonasal undifferentiated carcinoma (SNUC), grade 3 laryngeal chondrosarcoma, and high-grade spindle cell sarcoma. ^b^ Salivary gland, oropharynx, hypopharynx ^c^ Percentages calculated with respect to the total cohort (N=68) SCC: squamous cell carcinoma

Variable	Category	f (%) N=68
Diagnosis		
	Differentiated mod keratinizing SCC	30 (44.1)
	Other (SCC variants and sporadic variants^a^)	33 (48.5)
	Adenoid cystic carcinoma	3 (4.4)
	Nasal mucosal melanoma	2 (2.9)
Tumor Site		
	Oral cavity	29 (42.6)
	Larynx	21 (30.9)
	Nasal/Nasosinus	10 (14.7)
	Other^b^	8 (11.8)
Stage of cancer		
	I	11 (16.2)
	II	9 (13.3)
	III-IV	48 (70.6)
Treatment		
	Chemotherapy	1 (1.5)
	Surgical	12 (17.6)
	Neoadjuvant chemotherapy + Surgical	1 (1.5)
	Surgical + Adjuvant radiotherapy	11 (16.2)
	Surgical + Adjuvant radiotherapy/ chemotherapy	28 (41.2)
	Radiotherapy	4 (5.9)
	Radiotherapy/chemotherapy	11 (16.7)
Postoperative complications^c^		
	Fistulas (cutaneous, pharyngocutaneous, oronasal, tracheoesophageal, salivary)	6 (8.8)
	Wound or flap (dehiscence, infection, necrosis)	5 (7.4)
	Facial neurological (paresis, paralysis, branch injury, paresthesias)	3 (4.4)
	Major bleeding (carotid artery, internal maxillary artery)	2 (2.9)
	Others (muscle fibrosis, seroma, abscessed lymphadenitis, neuropathic pain)	3 (4.4)

Postoperative outcomes were available for most patients: 33 patients (48.6%) experienced no postoperative complications, whereas 19 (27.9%) had specific complications documented. In 16 patients (23.5%), postoperative outcome data were not recorded (Table 2).

For analytical purposes, patients were categorized into three treatment-based groups: surgery, radiotherapy, and chemotherapy. Each group comprised all patients who received the respective modality, whether as monotherapy or as part of multimodal therapy; therefore, the groups were not mutually exclusive (Table 3).

**Table 3:** Characteristics of the treatment and adjuvant received Data are expressed as frequencies and percentages in parentheses for categorical variables.

Variable	f (%)
Type of Treatment	
Surgical	52 (76.5)
Radiotherapy	54 (79.4)
Chemotherapy	41 (60.3)
Postoperative complications	19 (27.9)
Neoadjuvant/Adjuvant treatment	40 (58.9)
Type	
Chemotherapy	1 (2.5)
Radiotherapy + Chemotherapy	28 (70.0)
Radiotherapy	11 (27.5)

Baseline, end-of-treatment, and three-month HRQOL by domain

At baseline, Physical Well-Being (PWB) was the lowest-scoring domain, whereas Social/Family Well-Being (SWB) had the highest scores. At treatment completion, Emotional Well-Being (EWB) became the lowest-scoring domain while SWB remained the highest. At three months, PWB again showed the poorest scores, with EWB still low (Table 4).

**Table 4:** Comparison of quality of life and subscales before, after, and at three months after treatment Data are expressed as means and standard deviations. Each scale was transformed to a 0–100 range, with 100 indicating the best quality of life in each domain. Superscripts (a-c) denote the results of multiple comparisons across time points (Pre, Post, and 3 months) within each subscale. Means sharing the same letter are not statistically different, whereas different letters indicate a statistically significant difference (p < 0.05). A repeated-measures MANOVA was used, with sphericity assumed according to Mauchly’s test; for variables in which sphericity was not assumed, the Greenhouse–Geisser correction was applied. A p-value < 0.05 was considered statistically significant; F values correspond to ANOVA results. HNQ: Head and Neck Questionnaire; MANOVA: multivariate analysis of variance; ANOVA: analysis of variance

	Pre	Post	3 months	p-value	F-value
Physical Wellness	22.48 ± 20.03 ^a^	37.45 ± 22.43^b^	13.39 ± 17.65^ c^	<0.0001	37.18
Social Wellness	68.12 ± 16.78 ^a^	73.53 ± 15.27^b^	74.89 ± 15.98^ b^	<0.0001	10.97
Emotional Well-Being	36.03 ± 20.85^a^	27.21 ± 15.04^b^	22.79 ± 11.9^ c^	<0.0001	20.63
Functional Wellness	51 ± 22.57^ a^	43.17 ± 19.99^b^	64.6 ± 17.38^ c^	<0.0001	37.36
HNQ	34.93 ± 12.08^a^	31.16 ± 13.39^b^	34.96 ± 8.72^ a^	0.015	4.35
Global – Quality of Life	41.61 ± 8.04	41.44 ± 6.48	41.36 ± 5.88	0.958	0.043

Domain trajectories

**Figure 1:**
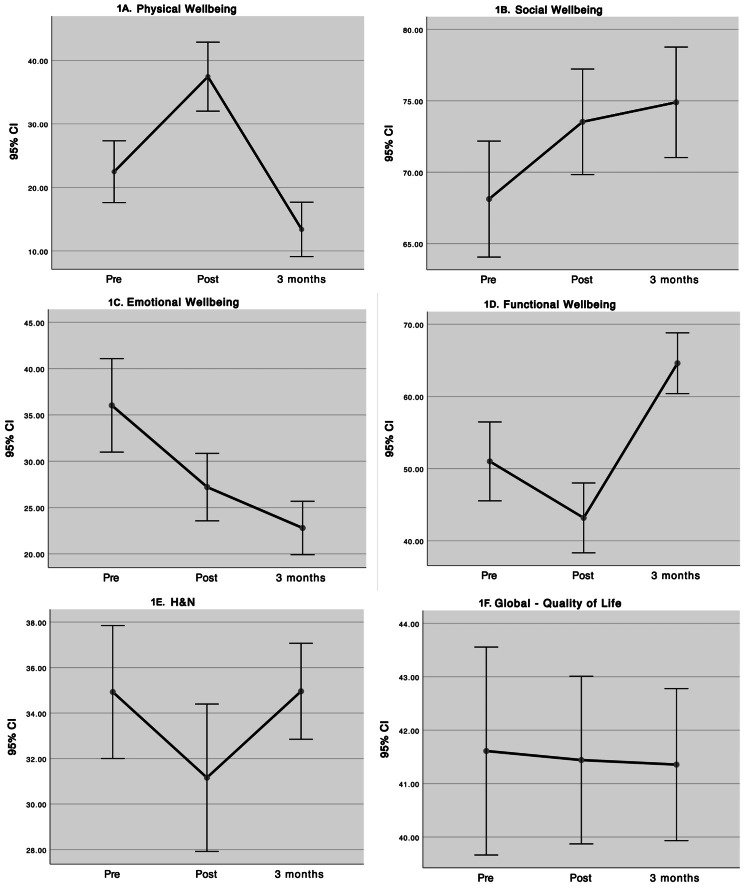
Comparison of quality of life and subscales before, after, and at three months after treatment. The FACT-H&N total score showed no significant differences across the three time points. 1A. Physical Well-Being; 1B. Social Well-Being; 1C. Emotional Well-Being; 1D. Functional Well-Being; 1E. Head and Neck; 1F. Global Quality of Life FACT-H&N: Functional Assessment of Cancer Therapy-Head & Neck

At baseline, Emotional Well-Being (EWB) exhibited the largest statistically significant decline across assessments; Social/Family Well-Being (SWB) increased after treatment and remained stable from one to three months. Physical Well-Being (PWB) improved at one month but fell below baseline by three months. Functional Well-Being (FWB) decreased at one month and improved by three months. The Head & Neck (H&N) subscale decreased at one month and returned to pretreatment values at three months. The FACT-H&N total score showed no significant differences across the three time points (Figure 1).

Associations by treatment modality

Surgery

**Figure 2:**
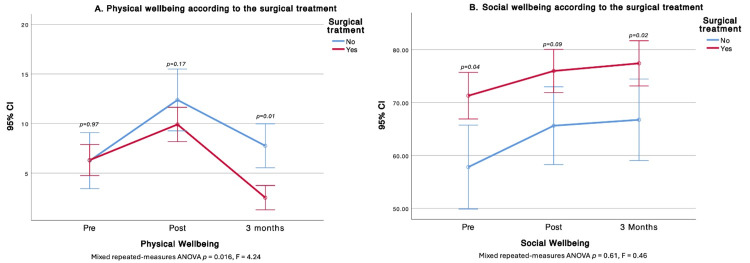
Comparison of Physical and Social Well-Being before, after, and at three months after surgical treatment A. Physical well-being according to the surgical treatment; B. Social well-being according to the surgical treatment

**Figure 3:**
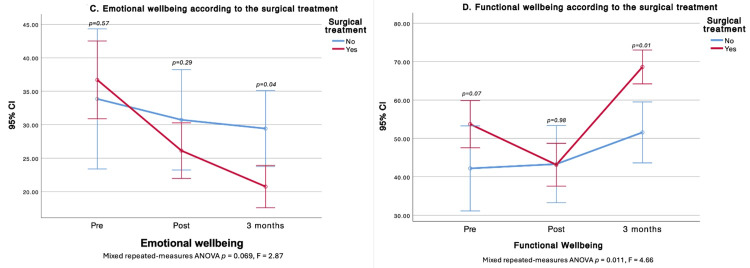
Comparison of Emotional and Functional Well-Being before, after, and at three months after surgical treatment C. Emotional well-being according to the surgical treatment; D. Functional well-being according to the surgical treatment

**Figure 4:**
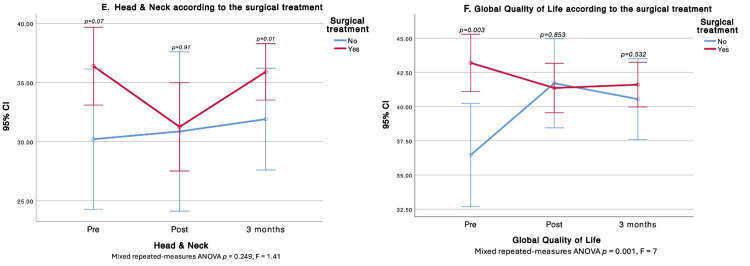
Comparison of Head & Neck and Global Quality of Life before, after, and at three months after surgical treatment E. Head & Neck according to the surgical treatment; F. Global quality of life according to the surgical treatment

Treatment type was associated with HRQOL differences. Surgery affected PWB, FWB, and the total score. At three months, non-operated patients had higher PWB, whereas operated patients demonstrated better FWB. Patients scheduled for surgery started with higher global HRQOL; however, by one and three months, total scores were similar between groups. Surgery did not alter the SWB trajectory over time, but cross-sectional differences favored the surgical group at baseline and at three months (higher SWB). In contrast, at three months, the operated group showed lower EWB (Figures 2-4).

Radiotherapy

**Figure 5:**
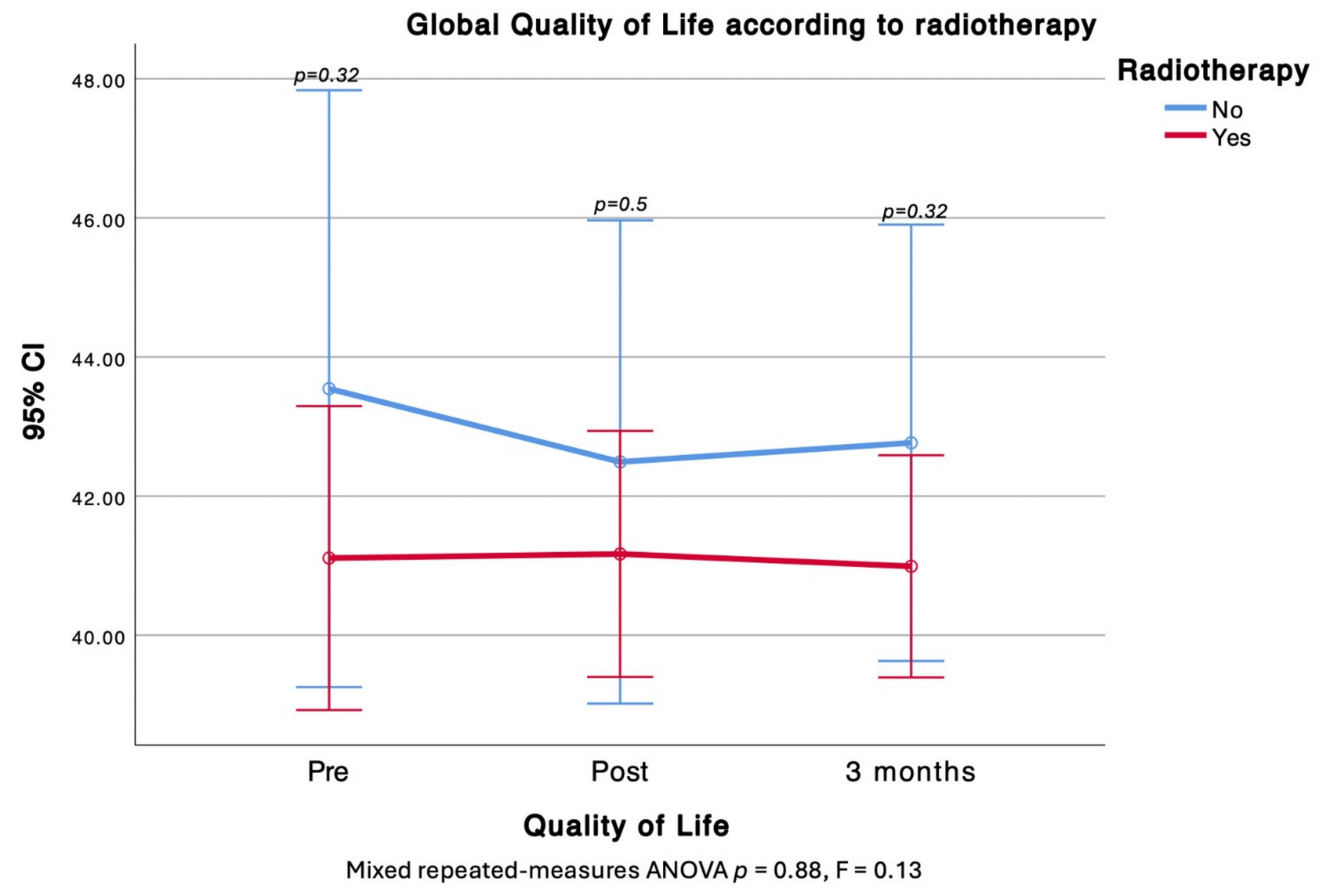
Comparison of Global Quality of Life before, after, and at three months of radiotherapy

Perceived HRQOL trajectories or subscales did not change over time. Patients slated to receive radiotherapy had worse baseline PWB and FWB, but no between-group differences were observed after treatment. The H&N subscale, however, was lower in those who received radiotherapy both immediately post-RT and at three months (Figure 5).

Chemotherapy

**Figure 6:**
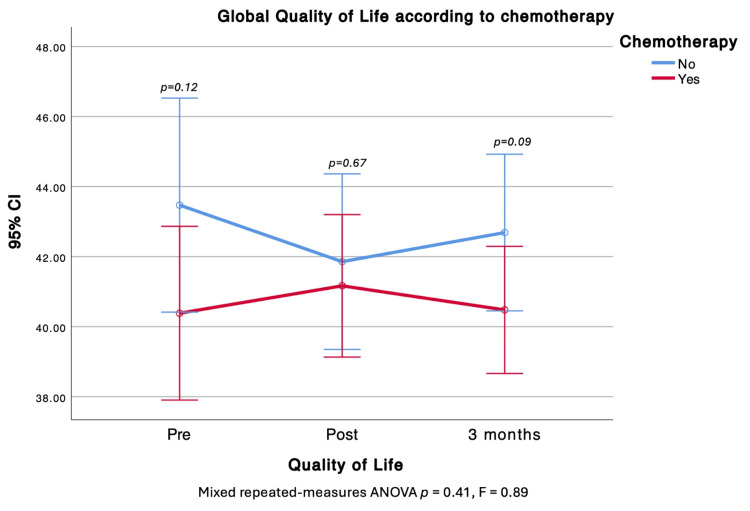
Comparison of Global Quality of Life before, after, and at three months of chemotherapy

Within-subject HRQOL trajectories were not influenced over time. Patients who received chemotherapy had higher PWB before and after treatment; in contrast, EWB and the H&N subscale were lower in the chemotherapy group at the analyzed time points (Figure 6).

## Discussion

HRQOL is a key therapeutic endpoint in upper aerodigestive tract (UADT) cancer. In our cohort, FACT-H&N scores improved significantly after treatment when surgery was part of care--both with and without adjuvant therapy--with the greatest gains observed in combined (multimodality) approaches. Nevertheless, meaningful limitations persisted (e.g., eating, communication, and residual symptom burden). Taken together, these findings support a positive contribution of oncologic treatment to perceived HRQOL, particularly when a surgical component is included.

Our results are consonant with those of Sujith et al., who documented frequent posttreatment functional challenges, most notably, altered salivary flow and difficulty eating, among UADT cancer survivors [12]. Despite these adverse effects, they observed partial improvements and highlighted the value of disease-specific instruments (e.g., FACT-H&N, QOL-OC) for capturing clinically relevant changes that can be diluted by generic scales [12]. In parallel, Martín Pérez et al. reported in a systematic review and meta-analysis that exercise-based rehabilitation improves muscular function and reduces fatigue but does not consistently translate into significant gains on global quality-of-life scales [13]. Juxtaposed with our data, this suggests that subjective well-being is shaped by multiple determinants beyond symptom control or functional performance alone.

Sensory sequelae also merit attention. Post-laryngectomy anosmia has been shown to negatively affect HRQOL, a sensory dimension that conventional instruments may underrepresent [14]. This aligns with our observation that certain domains (e.g., sensory function and communication) remain vulnerable even when global scores improve.

From an organizational standpoint, multidisciplinary team (MDT) care is pivotal. Prgomet et al. underscored the MDT’s role in comprehensive recovery for head and neck oncology patients [15]. Our findings mirror this perspective: patients managed with surgery complemented by adjuvant therapy, structured clinical follow-up, and nutritional and psychological support achieved better HRQOL, indicating that multidimensional care enhances not only clinical outcomes but also patient-centered metrics.

Survivor behavior and engagement matter. Saeidzadeh et al. described self-management approaches--“taking charge,” “living with it,” and “engaging as needed”--that directly influence perceived well-being [16]. This behavioral lens complements our results and argues for individualized, empowerment-oriented strategies (education, symptom self-management, and tailored rehabilitation) to sustain and amplify HRQOL gains after treatment.

In interpreting these findings, several limitations must be considered. First, we did not include an untreated control group of patients with UADT cancer, which would have provided a clearer comparator for the observed HRQOL changes; such a design was not ethically acceptable because withholding oncologic treatment is incompatible with current standards of care. Second, the sample was relatively small (n = 68) and derived from a single tertiary center, which may limit the precision and generalizability of our results. Third, the cohort was heterogeneous in terms of histopathologic diagnosis and treatment modality, potentially masking differences in HRQOL trajectories between specific tumor sites or treatment strategies. Fourth, follow-up was limited to three months after treatment completion, so late complications and long-term HRQOL trends could not be adequately characterized. Finally, the study did not include a systematic evaluation of structured rehabilitation (e.g., speech and swallowing therapy, physical rehabilitation) or of adherence to these interventions, and their potential impact on HRQOL therefore remains uncertain. These limitations should be taken into account when interpreting our findings and underscore the need for larger, multicenter studies with longer follow-up and integrated rehabilitation assessment.

## Conclusions

Taken together, our findings, consistent with international literature, indicate that health-related quality of life (HRQOL) in patients with upper aerodigestive tract cancer can improve substantially after treatment when care is delivered within a comprehensive, patient-centered framework. Nevertheless, persistent deficits in sensory function, speech, and psychosocial adjustment remain, underscoring the need for adjunct, domain-targeted interventions and structured follow-up protocols that extend beyond oncologic disease control.
